# Minimally invasive capsule-string device enables spatially resolved microbiome profiling across the upper gastrointestinal tract

**DOI:** 10.1080/19490976.2026.2675764

**Published:** 2026-05-19

**Authors:** Kathryn Garvey, J. Kirk Harris, Glenn T. Furuta, Kendra A. Occhipinti, Brandie D. Wagner, Joseph Fernandez, Mitchell VeDepo, Jennifer Fouquier, Emily B. Hill, Charles E. Robertson, Steven Ackerman, Robin Shandas

**Affiliations:** a Department of Bioengineering, University of Colorado Anschutz Medical Campus, Aurora, CO, USA; b Department of Pediatrics, Section of Pulmonology, University of Colorado Anschutz Medical Campus, Aurora, CO, USA; c Department of Pediatrics, Section of Pediatric Gastroenterology, Hepatology and Nutrition, Children’s Hospital Colorado, Aurora, CO, USA; d Department of Biostatistics and Informatics, Colorado School of Public Health, Aurora, CO, USA; e Department of Pediatrics, Section of Nutrition, University of Colorado Anschutz Medical Campus, Aurora, CO, USA; f Department of Medicine, Section of Infectious Diseases, University of Colorado Anschutz Medical Campus, Aurora, CO, USA; g Department of Biochemistry and Molecular Genetics, University of Illinois Chicago, Aurora, CO, USA

**Keywords:** Upper gastrointestinal tract, microbiome profiling, capsule-string device, region-specific microbiota, mucosal and luminal communities, minimally invasive sampling, spatial organization

## Abstract

Regional variation in the human gastrointestinal microbiome remains difficult to characterize because existing sampling methods either rely on invasive endoscopy or stool, which poorly reflects the upper gut. We evaluated a minimally invasive capsule-string device capable of collecting luminal and mucosal material from the esophagus, stomach, duodenum, and jejunum during natural transit. In healthy adults, compartment-level samples were anatomically localized using pH, bile staining, and string length, and microbial communities were profiled by 16S rRNA gene sequencing. The device was well tolerated and consistently recovered sufficient biomass from all upper GI regions. Distinct microbial signatures were evident across compartments, with the strongest differences observed between proximal (esophageal and gastric) and small-intestinal communities. Although the individual host exerted the dominant influence on the overall community structure, a reproducible regional signal persisted after accounting for between-person variation. These findings demonstrate that capsule-string sampling provides reliable access to spatially resolved upper GI microbiota without endoscopy. This approach enables more precise mapping of gut microbial organization in vivo and creates new opportunities for longitudinal, mechanistic, and disease-focused studies of host‒microbiome interactions in regions that have historically been inaccessible.

## Introduction

Microbial communities manifest differently across ecological niches of the body; structural and functional heterogeneity, including local physiology, within each gastrointestinal (GI) compartment, gives rise to regional differences in gut microbial populations.^1-10^ These local environments are shaped by many factors, such as genes and diet, resulting in highly variable microbial communities both within and across individuals.^11^^,^^12^ Such variations in the GI microbiota can either support or disrupt local physiological functions, including nutrient absorption and metabolism, making them key indicators of regional gut health.^1^^,^^2^^,^^5^^,^^11^^,^^13^^,^^14^

Despite their central role in digestion, metabolism, and disease, the upper GI compartments remain among the least understood regions of the human gut. Conditions such as gastroesophageal reflux disease (GERD), Barrett’s esophagus, H. pylori gastritis, peptic ulcer disease, gastroparesis, celiac disease, and small intestinal dysbiosis all originate in or are linked to the esophagus, stomach, or proximal small intestine.^2^^,^^6^^,^^9^ Evaluating these regions typically relies on endoscopy, which enables direct visualization and biopsy collection. However, endoscopy is invasive, resource-intensive, and poorly suited for repeated or large-scale sampling, particularly in healthy individuals. As a result, most human microbiome studies rely on stool, which provides scalability but offers limited insight into the regional composition and function of the upper GI tract.^15-17^

Independent comparisons reinforce this limitation: preliminary studies showed that string-derived upper GI samples are compositionally distinct from stool and recover taxa under-represented in feces, indicating that string sampling queries a different ecological niche and complements rather than duplicates fecal profiling.^17^ Prior work with shorter strings has also shown that esophageal microbiome profiles collected by this device closely match paired biopsies, demonstrating that string-derived samples capture authentic local community structure.^18^^,^^19^ Together, these findings support the capsule-string approach as both biologically informative and clinically relevant.

Beyond microbiome profiling, the capsule-string device has proven uniquely capable of accessing post-gastric contents. Early pharmaceutical studies demonstrated that strings could recover bile acids and metabolites, including in pediatric populations, showing feasibility for capturing otherwise hard-to-reach biliary secretions.^20-23^ These findings expand the device’s utility beyond microbial ecology into translational domains such as drug development, bile acid metabolism, and enterohepatic cycling, all areas of growing interest in gastroenterology.

These and other studies highlight a critical gap: the lack of a minimally invasive method to capture spatially distinct upper GI microbiome profiles across healthy and disease conditions^1^^,^^2^^,^^15^^,^^16^ To address this need, we adapted a clinically employed capsule-string device (EnteroTracker®, EnteroTrack LLC, Aurora, CO), previously validated in esophageal disease,^18^^,^^24-27^ to extend beyond the stomach and reach into the small intestine. This design provides continuous mucosal and luminal sampling along the upper GI tract, enabling anatomical localization by length, pH, and bile staining.

Here, we report a pilot study in healthy adults to evaluate: (1) the feasibility and tolerability of this minimally invasive capsule-string device for large-scale human studies and (2) its ability to capture microbial community information from distinct upper GI regions. By systematically comparing total bacterial load, alpha and beta diversity, and taxonomic composition across compartments, we demonstrate that this approach enables region-specific profiling within a strongly individualized microbiome, offering a practical alternative to endoscopy and a complement to stool sampling.

## Materials and methods

### The capsule-string sampling device

**Figure 1.**
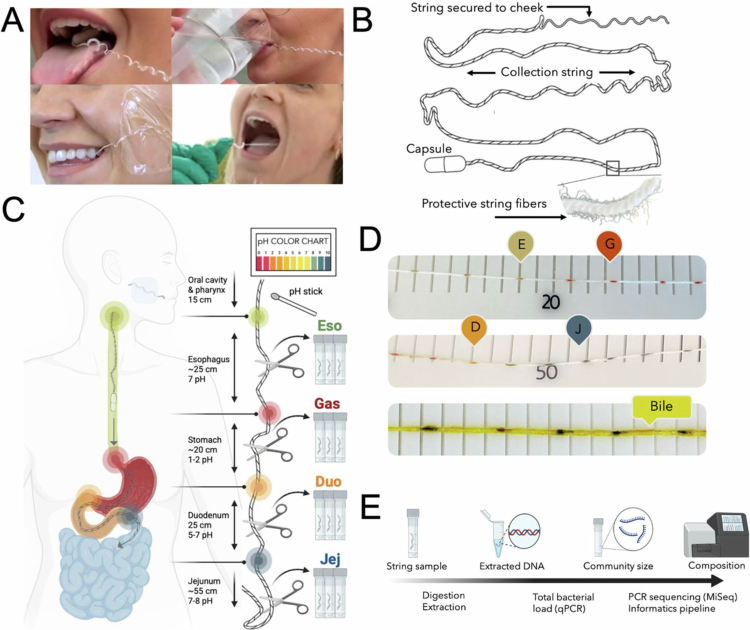
Capsule-string device and sample collection from the upper gastrointestinal (GI) tract. (A) Photos of device ingestion and string retrieval. (B) Schematic of the capsule-string device (EnteroTracker®) with an inset of the collection string. (C) Diagram of device deployment through the upper GI tract, with estimated regional lengths and pH ranges. A pH indicator stick is used post-retrieval to localize the digestive regions. Three 2-cm string segments are obtained from the proximal, middle, and distal portions of each compartment. (D) Retrieved string showing color changes from a pH indicator stick used to identify digestive segments. The bottom photo demonstrates bile staining. (E) String sample processing for quantification and sequencing of bacterial content. Panels C and E were created with BioRender.com.

The capsule-string sampling device (EnteroTracker®, EnteroTrack LLC, Aurora, CO; Figure 1B) used in this study was an extended-length research version of a clinically marketed capsule-string device. The standard clinical version is an FDA Class I, 510(k)-exempt device intended for the collection of upper gastrointestinal (GI) fluid/mucus in adult and pediatric patients. The extended-length version used here was developed from that device for research use in spatial microbiome profiling and was used under Institutional Review Board oversight as a non-significant risk device.

Relative to the standard 90-cm clinical device used in previous studies,^18^^,^^24-26^ the study device incorporated a 140-cm absorbent collection string and a proportionally larger capsule housing to accommodate the increased string length. In addition, the distal weighted ball bearing, which in the standard device is housed within the capsule, was adhered to the distal end of the extended string to facilitate distal progression during the longer dwell period. The string material and absorbent properties were otherwise unchanged.

The device consists of a weighted capsule containing an absorbent collection string wound inside. A small proximal portion of the string protrudes from the capsule; this end is held outside the mouth during swallowing and taped to the cheek. As the capsule transits the upper GI tract, the string unwinds. After capsule dissolution, the string remains in place for sampling and is subsequently retrieved through the mouth. The only procedural modification from the standard clinical protocol was extension of dwell time from 1 to 3 h to permit distal advancement into the proximal small intestine. No additional deployment or retrieval modifications were introduced.

### Study design

This study was approved by the Colorado Multiple Institutional Review Board (COMIRB; protocol #21-5118), which classified the capsule-string sampling device as a non-significant risk (NSR) device. Healthy adults aged 18–64 y were recruited. Eligibility criteria included the ability to comply with the pre-procedure fasting requirement and to swallow a pill-sized capsule. The exclusion criteria included known gastrointestinal disorders, gelatin allergy, antibiotic or proton pump inhibitor use within the prior 6 months, swallowing difficulties, and pregnancy. Written informed consent was obtained from all participants before enrollment.

Each participant swallowed one device with water after the required pre-procedure fast. Following ingestion, the string remained in situ for approximately 3 hours before retrieval by the study staff (Figure 1A). Upon retrieval, the string was placed on a clean high-density polyethylene board and evaluated for ordered physiologic transitions along its length. pH indicator markings were used to identify a proximal neutral segment consistent with esophageal exposure, followed by a clearly acidic gastric segment and then a distal rise in pH consistent with post-gastric progression; visible bile staining, when present, provided additional support for post-gastric localization (Figure 1C). These physiologic transitions were cross-referenced with the measured string position/length to conservatively assign sampling regions corresponding to the esophagus, stomach, duodenum, and jejunum.

To assess within-compartment variability, three 2-cm string segments were collected from the proximal, middle, and distal portions of each assigned upper-GI compartment and stored at −80 °C until processing. All participants completed a post-procedure survey following study completion (Supplemental Material, S1). Monitoring for adverse events that occurred during device administration and immediately after retrieval.

### Sample processing

Individual string segments were enzymatically digested prior to DNA extraction as previously described (see Figure 1E).^28-30^ DNA was extracted using the Qiagen EZ1 Tissue Kit or QIAamp PowerFecal Pro Kit (Qiagen, Germantown, MD) per manufacturer’s instructions. The extracted DNA was diluted 1:40 (TE buffer pH 8.0) and assayed in triplicate (4  μL template, dilution factor of 10) to determine the total bacterial load (TBL) using the assay described by Nadkarni et al.^31^

Bacterial profiles were determined by broad-range amplification and sequence analysis of 16S rRNA genes following previously described methods.^18^^,^^19^^,^^32-34^ In brief, amplicons were generated using primers that target approximately 300 base pairs of the V1V2 variable region of the 16S rRNA gene. The PCR products were normalized using agarose gel densitometry, pooled, lyophilized, purified, and concentrated using a DNA Clean and Concentrator Kit (Zymo, Irvine, CA). Pooled amplicons were quantified using a Qubit Fluorometer 2.0 (Invitrogen, Carlsbad, CA). The pool was diluted to 4 nM and denatured with 0.2 NaOH at room temperature. The denatured DNA was diluted to 15 pM and spiked with 25% of the Illumina PhiX control DNA prior to loading the sequencer. Illumina paired-end sequencing was performed on the MiSeq platform using a 500-cycle version 2 reagent kit.

Illumina MiSeq paired-end reads were aligned to the human reference genome Hg19 with bowtie2 and matching sequences discarded.^35^^,^^36^ The remaining non-human paired-end sequences were sorted by sample via barcodes in the paired reads with a Python script.^33^ Sorted paired-end sequence data were deposited in the NCBI Short Read Archive under accession number PRJNA1143534. The sorted paired reads were assembled using phrap.^37^^,^^38^ Pairs that did not assemble were discarded. The assembled sequence ends were trimmed over a moving window of 5 nucleotides until the average quality met or exceeded 20. Trimmed sequences with more than 1 ambiguity or shorter than 200 nucleotides were discarded. Potential chimeras, identified with Uchime (usearch6.0.203_i86linux32)^39^using the Schloss^40^ Silva reference sequences, were removed from subsequent analyses. The assembled sequences were aligned and classified with SINA (1.3.0-r23838)^41^ using the 418,497 bacterial sequences in Silva 115NR99^42^ as a reference, which was configured to yield the Silva taxonomy. Operational taxonomic units (OTUs) were produced by clustering sequences with identical taxonomic assignments. This process generated 14,987,841 sequences for 264 samples (average sequence length: 316 nt; average sample size: 56,772 sequences/sample; range 5125–278,910). The median Good’s coverage score was ≥ 99.65% at the rarefaction point of 5125. The software package Explicet (v2. 10.5, www.explicet.org)^43^ was used for initial data organization and exploratory visualization.

### Statistical analysis

Total bacterial load (TBL) was analyzed using a linear mixed-effects model fit to a repeated measures ANOVA to analyze the TBL values across different compartments. The ANOVA was fit to log_10_-transformed TBL values that included compartment and segment number as fixed effects, with random subject intercepts to account for individual variation between participants. Least square means (LSM) and Tukey-adjusted pairwise comparisons of the GI compartments and within-compartment segments (proximal, mid, and distal) were conducted to evaluate differences. Statistical significance was established a priori as *p* < 0.05. Confidence intervals were computed to indicate the uncertainty around the estimated bacterial load means.

Alpha diversity metrics, including the observed OTUs (Sobs), Shannon diversity index (H), and Shannon evenness (E), were calculated using the phyloseq R package. Differences in alpha diversity across GI regions (Eso, Gas, Duo, Jej) were assessed using the Kruskal–Wallis test, with pairwise differences examined using Dunn’s tests and Benjamini‒Hochberg correction. Beta diversity was calculated with vegdist using Morisita–Horn (counts), Bray‒Curtis (proportions), and Jaccard (presence/absence), and with GUniFrac and a Silva SSU 115 genus-level phylogenetic tree for weighted and unweighted UniFrac, and visualized by Non-metric Multidimensional Scaling (NMDS). Regional effects were tested by partial PERMANOVA (adonis2), linear-mixed-effects models (R packages lme4 and lmerTest), Tucker decomposition (R package Multiway), and partial distance-based redundancy analysis (dbRDA) (R package Vegan). To further quantify clustering, and within- vs between-group distances. Relative abundances were computed by transforming raw counts to proportions, and taxonomic composition was visualized using stacked bar plots at the phylum, order, and genus levels. For region-level comparisons, samples were merged by GI compartment and averaged across all participants. For individual-level views, replicates were aggregated by individual and compartment and visualized separately.

## Results

### Device feasibility and spatial sample recovery

All participants (100%) completed the procedure without adverse events and reported high tolerability. All participants felt safe using the capsule-string device and indicated that they would feel comfortable using it at home; 23 (96%) were willing to repeat the method, and 21 (88%) reported no concerns associated with device use. Eleven participants (46%) reported brief gagging or nausea lasting less than one hour. Three participants (12%) reported pre-procedure concern regarding swallowing but did not experience adverse outcomes (Table 1).

**Table 1.** Post-procedure survey results (*n* = 24).

Variable	Response		*N* (%)
Willing to repeat	Yes		23 (96)
	No		1 (4)
At-home use	Yes		24 (100)
	No		0 (0)
Felt safe	Yes		24 (100)
	No		0 (0)
Concerns	Yes		3 (12)
	No		21 (88)
Discomfort	None or little to none		21 (88)
Symptoms	None		8 (33)
	Nausea		5 (21)
	Duration	<1 h	5 (21)
	Severity	Mild to moderate	5 (21)
	Gagging		11 (46)
	Duration	<1 h	11(46)
	Severity	Mild to moderate	11 (46)

A total of 281 samples were recovered across all devices (24 participants × 3 segments × 4 compartments = 288 expected; 7 not recovered). One gastric segment was mishandled, and one string did not deploy into the small intestine. Bile staining was observed on 75% of collected string samples (Figure 1D), supporting successful small-intestinal deployment.

### A reproducible proximal-distal ecological gradient in bacterial load and alpha diversity

**Figure 2.**
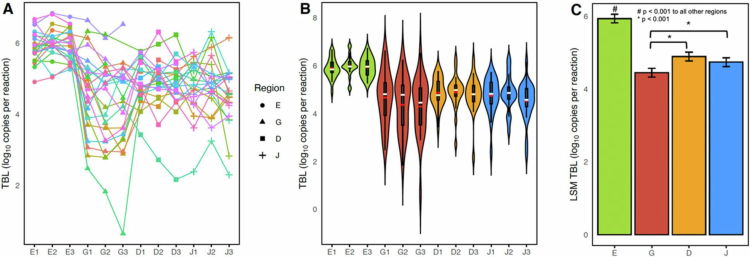
(A) Per-participant trajectories of TBL (log10 copies/reaction) across 12 ordered segments showed compartment-level shifts: highest in the esophagus, lowest in the stomach, with recovery in the small intestine. (B) Segment-level distributions are summarized on a log scale; median (white line) and mean (red line) are indicated. (C) Least square means (LSM ± SE) from repeated-measures ANOVA revealed significant differences across GI regions (Eso = 5.94 ± 0.12, Gas = 4.45 ± 0.12, Duo = 4.89 ± 0.12, Jej = 4.74 ± 0.12; all *p* < 0.001 except Duo vs Jej, n.s.). Proximal, mid, and distal segments within the compartments did not differ.

Per-participant trajectories of total bacterial load (TBL; qPCR-based 16S copy number) across the 12 ordered segments demonstrated a structured shift along the proximal–distal axis of the upper gastrointestinal tract (Figure 2A). Segment-level summaries across individuals (Figure 2B) showed that proximal, mid, and distal segments within the same anatomical compartment exhibited similar bacterial load, whereas clear differences were observed between major compartments. The bacterial load was highest in the esophagus and decreased in the stomach, with intermediate levels observed in the duodenum and jejunum.

We fit a linear mixed-effects model with compartment as a fixed effect and participant as a random intercept. The compartment had a significant fixed effect. The least-squares means (log10 copies/reaction ± SE) were esophagus (Eso) = 5.94 ± 0.12, gastric (Gas) = 4.45 ± 0.12, duodenum (Duo) = 4.89 ± 0.12, and jejunum (Jej) = 4.74 ± 0.12 (Figure 2C). Tukey-adjusted pairwise comparisons indicated that the esophagus differed significantly from all other compartments (all *p* < 0.001), and the duodenum differed from the esophagus and stomach (*p* < 0.001), whereas the duodenum and jejunum did not differ significantly. No significant differences were detected among the proximal, mid, and distal segments within the compartments, indicating intra-compartment stability.

**Figure 3.**
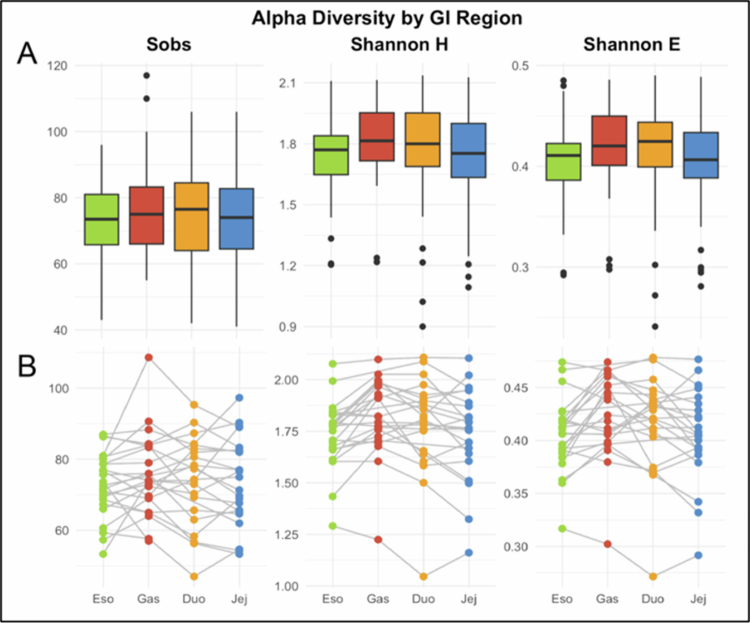
Alpha diversity of upper GI microbiota across regions: esophagus (Eso), stomach (Gas), duodenum (Duo), and jejunum (Jej). (A) Boxplots of observed richness (Sobs), Shannon diversity (H), and Shannon evenness (E). Richness did not differ (*p* = 0.76), whereas Shannon diversity (χ² = 9.28, *p* = 0.026) and evenness (χ² = 8.63, *p* = 0.035) varied by region. (B) Paired line plots show within-individual trajectories, reinforcing consistent esophagus–stomach shifts against a background of comparable richness across compartments.

Across the compartments, the alpha diversity metrics indicated that the differences in community structure were driven primarily by shifts in relative abundance rather than changes in overall richness (Figure 3A). The observed richness (Sobs) did not differ significantly by region (Kruskal–Wallis χ² = 1.16, *p* = 0.76), suggesting that similar numbers of taxa were detected in the esophagus, stomach, duodenum, and jejunum.

In contrast, the Shannon diversity (H) and Shannon evenness (E) differed significantly across the compartments (H: χ² = 9.28, *p* = 0.026; E: χ² = 8.63, *p* = 0.035). Post-hoc comparisons indicated higher diversity and evenness in the stomach relative to the esophagus, while other pairwise comparisons were not significant after correction for multiple testing. Regional differences in alpha diversity reflect redistribution in the relative abundances of taxa rather than large-scale gain or loss of taxa.

Within-individual trajectories (Figure 3B) demonstrated that these compartment-level patterns were detectable within participants, supporting that the observed differences were not solely attributable to inter-individual variability. The residual distributions showed comparable dispersion across regions, indicating consistent variability structure among the compartments. While the upper GI compartments harbor broadly overlapping taxonomic pools, their relative abundance structure varies by region.

### Host identity is the dominant signal, with consistent regional filtering

**Figure 4.**
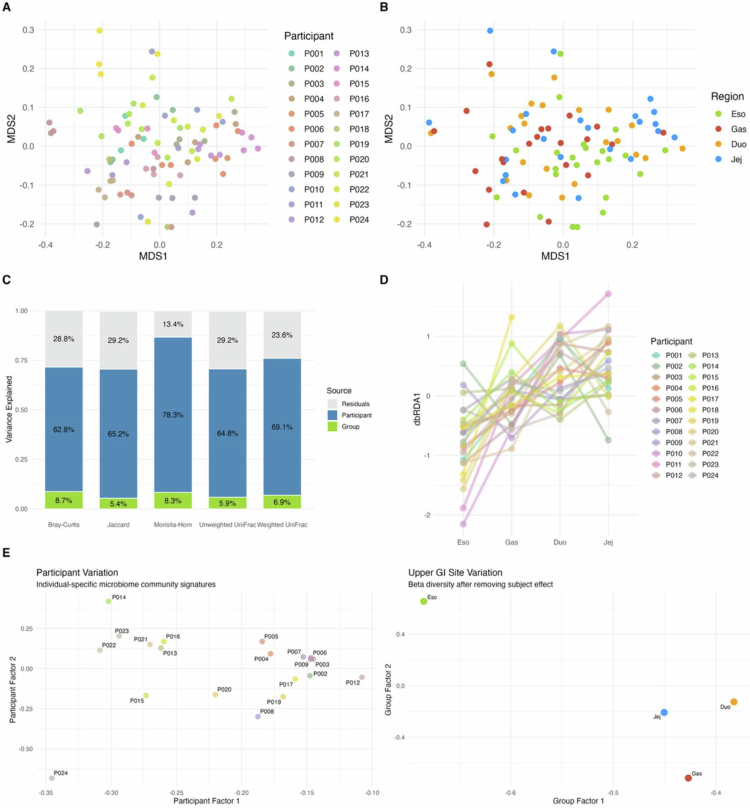
Beta diversity analysis of upper GI microbiome. Multidimensional scaling of weighted UniFrac distances colored by (A) participant (*n* = 24) and (B) gastrointestinal site: esophagus (Eso), stomach (Gas), duodenum (Duo), and jejunum (Jej). (C) Stacked bar charts showing the percentage of community variance attributed to the subject (blue), anatomical group (green), and residuals (grey) across non-phylogenetic distance metrics (Bray–Curtis, Jaccard, and Morisita–Horn) and phylogenetic distance metrics (weighted UniFrac and Unweighted UniFrac). Individual identity consistently explains the majority of variance, with a lesser portion attributed to the GI site. (D) Intra-individual spatial trajectories from partial distance-based redundancy analysis (dbRDA) showing microbial community shifts across anatomical sites. Participant-specific variance was accounted for to isolate spatial trends. The y-axis (dbRDA1) represents the variance constrained by anatomical position (esophagus to jejunum). Lines connect samples from the same participant (*n* = 24). (E) Ordination using Tucker decomposition to show individual-specific variation in microbial communities (left) and GI site (right) after statistically removing participant effects. Clear separation emerges between esophageal, gastric, and small intestinal sites.

Across non-phylogenetic beta diversity metrics (Bray–Curtis, Jaccard, and Morisita–Horn) and phylogenetic metrics (unweighted and weighted UniFrac), individual identity explained substantially more variation in community structure than anatomical region (Figure 4). Weighted UniFrac ordination plots illustrate broad clustering by participant (Figure 4A); when colored by GI site, the same ordination plots reveal partial but consistent compartment-level organization (Figure 4B). In variance-partitioned partial PERMANOVA models, subject identity explained 62.8–78.3% of the explainable variance, whereas GI site explained 5.4–8.7% for Bray–Curtis, Jaccard, Morisita–Horn, unweighted UniFrac, and weighted UniFrac (all *p* = 0.001; Figure 4C). The corresponding R^2^ values for the subject and region, respectively, were 0.628 and 0.087 for Bray–Curtis, 0.652 and 0.054 for Jaccard, 0.783 and 0.083 for Morisita–Horn, 0.648 and 0.059 for the unweighted UniFrac, and 0.691 and 0.069 for the weighted UniFrac (Figure 4C). Thus, although individual identity was the dominant driver of community composition, anatomical site remained a significant and reproducible contributor across all diversity metrics.

To separate the strong effect of host identity on beta diversity, Tucker decomposition was applied to isolate individual signatures from regional effects. After accounting for subject identity, clearer separation emerged among upper GI compartments, particularly between esophageal, gastric, and post-gastric communities, while duodenal and jejunal samples remained more similar to one another than to the proximal sites (Figure 4E).

Partial distance-based redundancy analysis (dbRDA) likewise identified a significant effect of GI site across all beta diversity metrics after accounting for participant effects (*p* < 0.029 for all metrics, F(1,66): Bray‒Curtis = 7.65; Jaccard = 6.12; Morisita‒Horn = 6.91; Unweighted UniFrac = 6.04; Weighted UniFrac = 4.12; Figure 4D). These trajectories indicate that within individuals, microbial communities shift reproducibly across anatomical regions even after the dominant subject effect is removed. However, linear mixed-effects models with site rank as a fixed effect and subject as a random effect revealed no significant progressive increase in dissimilarity from esophageal samples compared to gastric, duodenal, and jejunal samples, indicating that proximal-distal divergence does not follow a simple linear trajectory. Together, these findings indicate that upper GI microbial communities are structured by region, forming a spatially ordered gradient with compartment-specific differentiation.

### Environmental filtering across compartments and physiologic gradients

**Figure 5.**
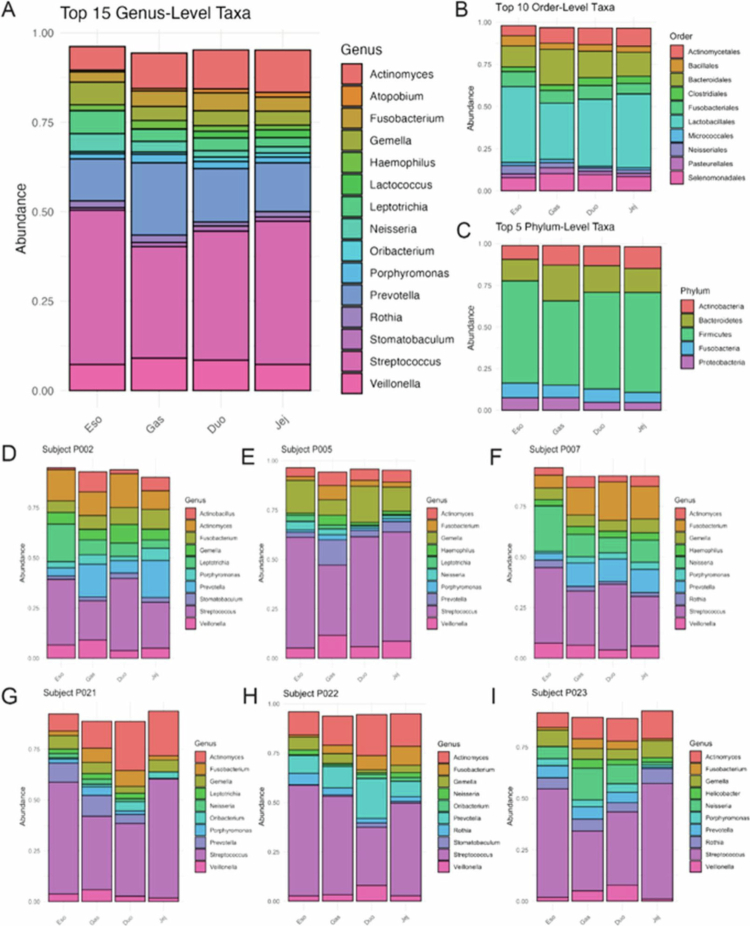
Taxonomic composition across gastrointestinal regions: esophagus (Eso), stomach (Gas), duodenum (Duo), and jejunum (Jej). Relative abundances of bacterial taxa averaged across all participants (*n* = 24) at the genus (A), order (B), and phylum (C) levels. Firmicutes and Bacteroidetes predominated, with additional contributions from Actinobacteria and Proteobacteria, varying by compartment. Individual-level genus profiles (D–I) highlight inter-individual variability alongside regional trends. Across all samples, the top 10 genera (e.g., Prevotella, Veillonella, Streptococcus, Actinomyces) accounted for a mean of 89.8% of the community composition (median 91.3%, IQR 87.8–93.7%), with regional means of Eso 92.5%, Gas 89.8%, Duo 88.5%, and Jej 87.9%.

Across individuals, the top 10 genera accounted for approximately 90% of total relative abundance (mean 89.8%, median 91.3%), indicating that a relatively small number of dominant taxa structured most of the community composition. Although additional taxa were detected at lower abundance, compartment-level differences were driven primarily by shifts in the relative abundances of shared dominant taxa rather than wholesale taxonomic replacement (Figure 5).

At broader taxonomic levels, Firmicutes and Bacteroidetes predominated across all compartments, with contributions from Actinobacteria and Proteobacteria throughout the upper GI tract (Figure 5B, C). Although these phyla were consistently detected, their proportional representation shifted across anatomical regions. At finer taxonomic resolution, dominant genera, including *Streptococcus*, *Veillonella*, *Prevotella*, and *Actinomyces,* were observed across multiple compartments, but their relative contributions varied systematically by region (Figure 5A). The esophagus was enriched for genera commonly associated with the oral microbiome, whereas gastric samples showed a more even structure. The duodenal and jejunal communities were more similar to one another than to proximal compartments, which is consistent with a gradual post-gastric compositional transition.

Selective shifts were particularly evident after gastric transit. Oral-associated genera such as *Rothia*, *Fusobacterium*, and *Leptotrichia* declined sharply in the stomach and remained at lower relative abundance distally. *Streptococcus*, the dominant esophageal genus, also decreased in the stomach before partially recovering in the small intestine. Together, these patterns support a model of selective ecological filtering across physiologic transitions, with the stomach functioning as a key bottleneck between oral-influenced and post-gastric microbial assemblages.

## Discussion

This initial study demonstrates that the capsule-string device can safely and reproducibly recover spatially resolved microbial communities across the proximal upper gastrointestinal tract. Across total bacterial load, alpha diversity, beta diversity, and taxonomic composition, we observed a consistent proximal-to-distal ecological gradient layered on a strong host-specific microbiome structure. The largest transitions occurred between esophageal/gastric compartments and the small intestine, whereas duodenal and jejunal communities were comparatively similar. At the same time, individual identity accounted for more variation than anatomical region, indicating that each participant carried a personalized microbial baseline on which reproducible compartment-level differences were superimposed. Additional larger-scale studies and direct comparison to biopsy or aspiration data still need to be performed, but these findings appear to support the capsule-string as a practical tool for mapping upper-GI microbial organization in vivo without endoscopy and for studying regional biology that is not accessible through stool alone.

Although this should be considered an early-stage, limited study, certain insights may be derived around the regional biogeography of the upper GI system. Regional patterns observed align with known upper-GI physiology and prior direct-sampling studies. The esophagus appears to function as an oral-facing and immunologically active interface, with the highest bacterial load and enrichment of genera commonly associated with the oral cavity, including *Streptococcus*, *Veillonella*, *Prevotella*, and *Actinomyces*. This is consistent with prior esophageal string- and biopsy-based studies showing that the esophageal microbiome is distinct from oral and nasal communities while remaining enriched for oral-associated taxa.^18^ Deviations from this baseline may herald discordance with local homeostasis as seen in conditions such as Eosinophilic Esophagitis or GERD.^19^ The results from the gastric region, in contrast, showed markedly lower bacterial load but relatively greater diversity and evenness than the esophagus, which is consistent with a more selective acid-filtered niche that reduces total biomass while reshaping the relative abundance of shared upper GI taxa.^6^^,^^44^ Distally, the duodenum and jejunum were most similar to one another in both bacterial load and beta diversity, which is consistent with previously found post-gastric conditions, including bile exposure, nutrient flow, rapid transit time, and changing oxygen tension.^2^^,^^45^^,^^46^ Importantly, the regional signal was not driven by wholesale taxonomic replacement, but rather by redistribution among dominant shared taxa, supporting a model in which local physiologic conditions generate reproducible compartment-specific ecological filtering across the proximal upper GI tract.

Despite the clear regional organization observed across the upper GI tract, inter-subject differences remained the dominant source of microbiome variation. Across both non-phylogenetic and phylogenetic beta-diversity frameworks, subject-to-subject differences contributed substantially more variance than anatomical site, indicating that each individual carried a strong personalized microbial baseline on which compartment-level differences were superimposed. At the same time, regional effects remained reproducible across metrics, and participant-adjusted analyses clarified separation between esophageal, gastric, and post-gastric communities, with duodenal and jejunal samples remaining more similar to one another than to proximal sites. This pattern is consistent with prior mucosal microbiome studies showing that inter-individual variation can exceed local site-to-site differences even when anatomically structured regional signatures remain detectable.^47^ Rather than diminishing the importance of spatial organization, this hierarchy helps clarify how upper GI microbiomes should be interpreted: region-specific structure should be evaluated as this may provide important clues regarding local disruptions in homeostasis, but local structure is embedded within a strong individualized background. This has important implications for study design, because within-person and longitudinal sampling may be more informative than purely cross-sectional comparisons when the goal is to detect meaningful regional change or intervention effects in the proximal gut.

A further consideration for continuous string-based sampling is the potential for retrieval-related contamination across adjacent compartments. The design and process features of the string device reduce contamination during retrieval, and several features of the present dataset argue against large-scale redistribution as the dominant explanation for the observed spatial patterns. The retrieved strings consistently showed sharp, ordered pH transitions and discrete bile staining rather than progressive homogenization, suggesting preservation of compartment-linked physiologic boundaries during withdrawal. In addition, the observed compartment-specific differences in bacterial load, beta diversity, and taxonomic composition would be difficult to reconcile if extensive mixing occurred across regions during retrieval. Nonetheless, because anatomical localization was operationally defined using physiologic markers rather than confirmed by concurrent imaging or direct comparator sampling, some degree of redistribution cannot be completely excluded and should be evaluated further in future validation studies, ideally against invasive collection methods such as biopsy.

At the taxonomic level, our findings were more concordant with prior upper GI direct-sampling studies than with stool-based profiles. Although *Firmicutes* and *Bacteroidetes* predominated broadly across compartments, the communities were enriched for upper GI-associated genera including *Prevotella*, *Veillonella*, *Streptococcus*, and *Actinomyces* rather than the strict anaerobic, colon-associated taxa that typically dominate feces.^1^^,^^2^^,^^6-9^^,^^18^ This distinction is not surprising, given that stool primarily reflects pooled distal luminal communities, whereas upper GI approaches interrogate more proximal and spatially restricted ecological niches.^1^^,^^2^^,^^5^ In paired small-bowel and stool comparisons, duodenal communities have likewise been shown to differ compositionally from feces and other upper GI platforms, including biopsy-, aspirate-, and capsule-based methods, which have similarly recovered proximal taxa that are underrepresented or absent in stool.^3^^,^^4^^,^^8^^,^^9^ Endoscopic sampling remains invasive and poorly suited to repeated use in healthy volunteers or longitudinal studies, while ingestible capsule systems such as SIMBA provide access to more distal sites but capture only a limited, localized sample.^3^^,^^4^^,^^15^^,^^16^ Viewed in this context, the capsule-string is best understood not as a replacement for stool or endoscopy, but as a complementary approach that helps bridge the gap between scalable fecal profiling and invasive direct sampling by enabling repeated, regionally resolved access to the proximal gut.

The main clinical and translational value of this approach appears to be not simply that it samples the upper GI tract, but that it can do so repeatedly, with compartment-level resolution in a format better suited to healthy volunteers and longitudinal designs than endoscopy. This creates opportunities to track region-specific microbial changes in settings where stool may be an insensitive surrogate, including esophageal disorders, gastric inflammation, proximal small-intestinal dysbiosis, and nutrition intervention studies. As such, the capsule-string complements rather than replaces stool and biopsy: stool remains valuable for distal lumen ecology at scale, and biopsy remains essential when histology or tightly localized mucosal pathology is required, whereas the capsule-string likely fills a practical gap for repeated, minimally invasive, regionally resolved upper GI sampling.

This study also has important limitations. First, anatomical localization was operational rather than confirmed by concurrent imaging or endoscopy, although pH transitions, string length, and bile staining together provided a practical framework for conservative compartment assignment. Second, the cohort was modest and limited to healthy adults at a single timepoint, so these data should be interpreted as an initial normative baseline rather than a comprehensive map of upper-GI variability. Formal upper GI symptom phenotyping was not performed, limiting the assessment of subtle symptom-associated variation. Also, taxonomic profiling relied on 16S rRNA gene sequencing, which limits strain-level resolution and functional inference. Finally, the study did not include direct comparison with established upper GI sampling approaches, such as aspirate or endoscopic biopsy, and relatively few healthy, multi-compartment upper GI microbiome datasets exist for cross-platform benchmarking. Future studies incorporating orthogonal localization, direct comparator sampling, richer clinical phenotyping, and longitudinal repeat sampling will be important to further validate and extend these findings.

In summary, these initial findings suggest that string-based spatial sampling may represent a useful methodology for human subjects research: it allows scalable collection of the upper-GI microbiome, and spatial variations to be interpreted in their native anatomical contexts. In practice, this means that compartment-specific abnormalities, such as unexpectedly high gastric biomass, altered proximal diversity, or loss of the usual similarity between the duodenum and jejunum, could be detected directly rather than inferred indirectly from stool. As multi-omics methods mature, pairing spatially resolved microbiome data with bile acids, metabolites, host proteins, or inflammatory markers may help define functional signatures of proximal gut health that are currently invisible in fecal studies. In that sense, the capsule-string may enable a shift from treating the gut microbiome as a single pooled ecosystem toward viewing it as a set of connected but distinct ecological niches, each with its own relevance to physiology and disease.

## Supplementary Material

Supplemental Material.docx Supplemental Material.docx

## Data Availability

Raw sequencing data have been deposited in the NCBI Sequence Read Archive (SRA) under BioProject accession PRJNA1143534.
