# Supplementary material for: Minimally invasive capsule-string device enables spatially resolved microbiome profiling across the upper gastrointestinal tract
Source: Gut Microbes. 2026 May 19;18(1):2675764. doi: 10.1080/19490976.2026.2675764 (PMC13196630; doi:10.1080/19490976.2026.2675764)
Supplement: Supplemental Material.docx [file KGMI_A_2675764_SM5781.docx]

**Supplemental Material**

S1. Tolerability questionnaire.

Please answer the following questions about your experience with the EnteroTracker® (ETK), which is the small capsule-based device with the string that you swallowed for a short period.

1. Would you be willing to repeat the ETK?
   1. Yes
   2. No
   3. Don’t know/not sure
2. Did you experience any discomfort with the ETK?
   1. No discomfort
   2. Little to no discomfort
   3. Moderate discomfort
   4. Moderate to severe discomfort
   5. Don’t know/not sure
3. From your experience, do you feel you could perform the ETK at-home?
   1. Yes
   2. No
   3. Don’t know/not sure
4. Overall, how would you rate your experience with the ETK?
   1. Very poor              b. Poor           c. Neutral        d. Good           e. Great
5. I felt safe swallowing the ETK:
   1. Yes
   2. No
6. I was concerned about swallowing the ETK:
   1. Yes
   2. No
7. If yes, what concerns did you have? __________________________________________________
8. What symptoms/side-effects did you experience during the ETK?

|  | Yes | No | If yes, length of symptoms (in hours) | If yes, how would you describe the severity of these symptoms?? |
| --- | --- | --- | --- | --- |
| None |  |  |  |  |
| Nausea |  |  |  | Mild discomfort  Moderate discomfort  Severe discomfort |
| Choking/Gagging |  |  |  | Mild discomfort  Moderate discomfort  Severe discomfort |
| Sore throat |  |  |  | Mild discomfort  Moderate discomfort  Severe discomfort |
| Vomiting |  |  |  | Mild discomfort  Moderate discomfort  Severe discomfort |
| Chest pain |  |  |  | Mild discomfort  Moderate discomfort  Severe discomfort |
| Abdominal pain |  |  |  | Mild discomfort  Moderate discomfort  Severe discomfort |
| Other  Please Specify: |  |  |  | Mild discomfort  Moderate discomfort  Severe discomfort |

**Supplemental Table 1.** Post-procedure survey results (n=24).

| Variable | Response | | N (%) |
| --- | --- | --- | --- |
| Willing to repeat | Yes | | 23 (96) |
|  | No | | 1 (4) |
| At-home use | Yes | | 24 (100) |
|  | No | | 0 (0) |
| Felt safe | Yes | | 24 (100) |
|  | No | | 0 (0) |
| Concerns | Yes | | 3 (12) |
|  | No | | 21 (88) |
| Discomfort | None or little to none | | 21 (88) |
| Symptoms | None | | 8 (33) |
|  | Nausea | | 5 (21) |
|  | Duration | <1 hour | 5 (21) |
|  | Severity | Mild to moderate | 5 (21) |
|  | Gagging |  | 11 (46) |
|  | Duration | <1 hour | 11(46) |
|  | Severity | Mild to moderate | 11 (46) |
